# The complete chloroplast genome of *Pinus yunnanensis* Franchet (Pinaceae)

**DOI:** 10.1080/23802359.2019.1565929

**Published:** 2019-07-16

**Authors:** Jing Qiu, Lin Chen, Xiangui Yi, Mingzhi Li

**Affiliations:** a Institute of Architecture, Sanjiang University, Nanjing, China;; b Co-Innovation Center for Sustainable Forestry in Southern China, Nanjing Forestry University, Nanjing, China;; c College of Biology and Environment, Nanjing Forestry University, Nanjing, China;; d Genepioneer Biotechnologies Co. Ltd, Nanjing, China

**Keywords:** *Pinus yunnanensis*, complete chloroplast genome, phylogenetic analysis, Pinaceae

## Abstract

*Pinus yunnanensis* is distributed in Southwestern China which is one of the main forest tree species. It is used for construction, railway sleepers, boards, furniture, and wood pulp. Due to the genetic infiltration hybridization between *P. yunnanensis* with *P. densata* and *P. kesiya*, the classification boundary between these groups is blurred and the identification is difficult. In this study, the complete chloroplast (cp) genome sequence of *P. yunnanensis* was determined using next-generation sequencing. The entire cp genome was determined to be 119,707 bp in length. It contained large single-copy (LSC) and small single-copy (SSC) regions of 65,619 and 53,098 bp, respectively, which were separated by a pair of 495bp inverted repeat (IR) regions. The genome contained 116 genes, including 76 protein-coding genes, 36 tRNA genes, and 4 rRNA genes. The overall GC content of the genome is 38.5%. A phylogenetic tree reconstructed by 23 chloroplast genomes reveals that *P. yunnanensis* is most related with *P. kesiya.*

The genus *Pinus* Linn. comprises approximately 80 species and occurs widely throughout the northern hemisphere. There are about 22 species of *Pinus* in China, which are widely distributed and are one of the important afforestation species (Fu et al. 1999). *Pinus yunnanensis* Franchet is distributed at altitudes between 400 m and 3100 m on mountains, river basins, dry and sunny slopes in Southwestern China which is one of the main forest tree species. It is mainly used for construction, sleepers, boards, furniture, and wood pulp, while it also can be a source of resin and eatable fungi producing. Pine needle oil can be extracted from pine needles. Due to the genetic infiltration, hybridization between *P. yunnanensis* with *P. densata* Masters and *P. kesiya* Royle ex Gordon, the classification boundary between these groups is blurred and the identification is difficult (Yu et al. 2000). So, it is necessary to develop genomic resources for *P. yunnanensis* to provide intragenic information for clarifying the taxonomic identities and valuable information about the course of evolution of *Pinus*.

The total genomic DNA was extracted from the fresh leaves of *P. yunnanensis* (Tianchi Nature Reserve of Yunlong County, 25.87°N, 99.28°E) using the DNeasy Plant Mini Kit (Qiagen, Valencia, CA, USA). The DNA was stored at -80 °C in our lab. The whole genome sequencing was conducted by Nanjing Genepioneer Biotechnologies Inc. (Nanjing, China) on the Illumina Hiseq platform. The filtered sequences were assembled using the program SPAdes assembler 3.10.0 (Bankevich et al. 2012). Annotation was performed using the DOGMA (Wyman et al. 2004) and BLAST searches. The plastome of *P. yunnanensis* was determined to comprise double-stranded, circular DNA of 119,707 bp containing two inverted repeat (IR) regions of 495 bp each, separated by a large single-copy (LSC) and small single-copy (SSC) regions of 65,619 and 53,098 bp, respectively (NCBI acc. no. MK007968). The genome contained 116 genes, including 76 protein-coding genes, 36 tRNA genes, and 4 rRNA genes. One tRNA gene trnI-CAU was duplicated in the IR region. Most of the genes occurred as a single-copy, while two PCGs (psaM and psbA) and six tRNA (TrnI-CAU, trnG-GCC, trnH-GUG, trnL-UAA, trnS-GCU, and trnT-GGU) had two copies, respectively. In addition, two PCGs (rps12 and ycf3) had two introns each, six PCGs (atpF, petB, petD, rpl2, rpl16, and rpoC1) and six tRNA genes (trnA-UGC, trnG-GCC, trnI-GAU, trnK-UUU, trnL-UAA, and trnV-UAC) contained one intron. The overall GC content of *P. yunnanensis* cp genome is 38.5% and the corresponding values in LSC, SSC, and IR regions are 37.9, 39.4, and 36.4%, respectively.

To investigate its taxonomic status, alignment was performed on the 23 chloroplast genome (22 Asian Pinus species and the outgroup *Picea asperata* Masters) sequences using MAFFT v7.307 (Katoh and Standley 2013), and a maximum likelihood (ML) was constructed by FastTree version 2.1.10 (Price et al. 2010). The ML phylogenetic tree shows that *P. yunnanensis* formed a strongly-supported clade with *P. kesiya* and *P. densata* (Figure 1).

**Figure 1.**
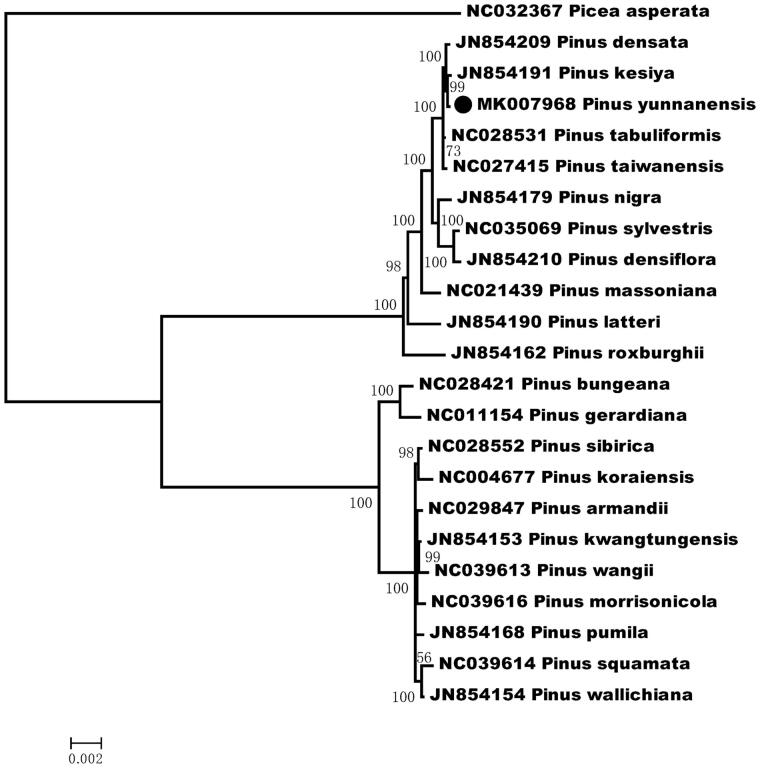
Phylogenetic tree inferred by Maximum Likelihood (ML) method based on the complete chloroplast genome of 22 species of Asian Pinus, bootstrap values (%) are shown on the branch.
